# Co-fermentation involving *Lysinibacillus* sp. and *Aspergillus flavus* for simultaneous palm oil waste treatment and renewable biomass fuel production

**DOI:** 10.3934/microbiol.2022025

**Published:** 2022-09-16

**Authors:** Nurul Alia Syufina Abu Bakar, Nur Aliyyah Khuzaini, Siti Baidurah

**Affiliations:** School of Industrial Technology, Universiti Sains Malaysia, Minden 11800, Penang, Malaysia

**Keywords:** palm oil mill effluent, co-fermentation, biological treatment, *Lysinibacillus* sp., *Aspergillus* sp.

## Abstract

Biomass fuel is one of the renewable energy sources that can be produced by valorization of palm oil mill effluent (POME) and empty fruit bunch (EFB). POME and EFB are available abundantly in Malaysia and only small portion is utilized to produce other value-added products. The objective of this study is to: (1) utilize the wastes from agro-industrial sector especially palm oil wastes and bio-valorize into value-added product of biomass fuel with high CEV, and simultaneously (2) reduce the waste accumulated in the palm oil factory. In this study, co-fermentation of bacteria (*Lysinibacillus* sp.) and fungus (*Aspergillus flavus*) at 37 °C, 180 rpm for 5 days, followed by overnight oven-dry at 85 °C was conducted utilizing a mixture of POME and EFB with the ratio of 7:3 at laboratory scale. Three fermentation medium conditions were performed, namely: (1) Group 1: autoclaved POME and EFB without addition of any microorganisms, (2) Group 2: autoclaved POME and EFB with the addition of *Lysinibacillus* sp. LC 556247 and *Aspergillus flavus*, and (3) Group 3: POME and EFB as it is (non-sterile). Among all condition, Group 2 with co-fermentation evinced the highest calorific energy value (CEV) of 26.71 MJ/kg, highest biochemical oxygen demand (BOD) removal efficiency of 61.11%, chemical oxygen demand (COD) removal efficiency at 48.47%, and total suspended solid (TSS) reduction of 37.12%. Overall, this study successfully utilized abundant POME and EFB waste and turn into value added product of renewable biomass fuel with high CEV percentage and simultaneously able to reduce abundant liquid waste.

## 1. Introduction

The palm oil industry has made a huge contribution to economic development in Malaysia as the oil derived from the palm fruit is easy to extract and applicable as a raw material for the production of various daily products such as oil and soap [1],[2]. However, the proliferation of palm oil has posed a massive environmental threat. Palm oil mill effluent (POME) and empty fruit bunch (EFB) are the waste that is generated from the production of palm oil may cause severe pollution if left in their natural state without any treatment [3]. In 2005, 14.8 million tonnes of crude oil palm were produced in Malaysia and the average of POME released to the environment is 53 million m^3^ [4]. The number of POME waste generated was increased in 2013, whereby 44 million m^3^ POME from 19.66 million tons of total crude palm oil [3]. Averagely, *circa* 2.5 to 3.5 tonne of POME is produced every year in Malaysia [5]. Moreover, 7 million tons of EFB was recorded in 2007 and the conventional solution to eliminate this waste is through the burning process [6]. The utilization of various industrial wastes for production of other value-added products is deem practical and has been delve deeply by many researchers [7]–[12].

POME is known as a high potential material for bioenergy production such as biogas due to it comprised of myriad organic contents [13]. EFB also can be utilized for biogas production but it requires pre-treatment to enhance the production yield [14]. Increment of 25–32% methane production was observed from the co-digestion of EFB and POME [15]. Biological processing methods in the presence of both bacteria and fungus can be applied for the valorization and remediation of POME and EFB for value-added products focusing on the production of biomass fuel. Octiva et al. (2018) conducted an experiment by mixing POME and EFB with the ratio of 30:1 in a bioreactor with the presence of thermophilic bacteria [16]. The highest gas production of 80.30 L/mg and the highest methane content of 80.10% was successfully achieved [16]. In other recent research conducted by Mohammad et al., the highest calorific energy value (CEV) of 21.25 ± 0.19 MJ/kg, was achieved when POME was treated with *Lysinibacillus sp*. LC 556247 after 48 hours of fermentation [17]. However, the aforementioned biological treatment method can still be improved to increase the CEV by utilizing co-fermentation of various microorganisms. High CEV value is an indicator to portray high quality of biofuels.

The compactness and low porosity are among the essential aspects to produce a high-quality biomass fuel pellet. It is worth noting that EFB biomassic pellet exhibited high degree of compactness, high mechanical durability, and low internal porosity [18]. This is due to the fibrous material such as EFB consists of naturally occurring composites fibres which primarily comprise of rigid, crystalline cellulose microfibrils which are embedded in a soft, amorphous matrix of hemicellulose and lignin [19]. Volatile matter makes up the majority of EFB fibres (up to 83.36%), followed by fixed carbon (18.3%), moisture (14.8%), and ash (up to 13.65%) [20]. The high volatile matter composition of EFB fibres suggests their ease of igniting and catch fire [19]. Since EFB fibres contain high amount of fixed carbon, significant amount of heat can be generated upon combustion in a boiler to generate energy [20].

In this study, the effects of fermentation with and without the presence of *Lysinibacillus* sp. LC 556247 and *Aspergillus flavus* on the production of high CEV biofuels were explored by valorizing POME and EFB, which also serves as feedstocks, at laboratory scale using shake flask. Both bacteria and fungi have been isolated from POME and are thus regarded to be resilient microorganisms that can thrive in extreme conditions [17]. These microbes have the potential to biodegrade the complex organic compounds to the simpler form. The potential for valorizing mixture of POME and EFB into useful energy sources is touted as practical and prevalent treatment since these resources are abundant wastes from the oil palm plantation. The novelty of this study is to elucidate the effects of co-fermentation on enhancing the biodegradation of organic matter and simultaneously increase the CEV. In this study, three fermentation conditions were performed using the mixture of POME and EFB in the ratio of 7:3, *viz*.: (1) Group 1: autoclaved POME and EFB without addition of any microorganisms, (2) Group 2: autoclaved POME and EFB with the addition of *Lysinibacillus* sp. LC 556247 and *Aspergillus flavus*, and (3) Group 3: POME and EFB as it is (non-sterile). The agitation speed and temperature were set at 180 rpm and 35 ± 2 °C, respectively for all three fermentations condition. Fermentation was conducted for five consecutive days followed by oven-drying procedure. The CEV of the resulting pellet was elucidated in order to identify the ideal and optimum fermentation condition to produce a renewable energy source, *viz*. biomass fuel. In addition, moisture content (MC), biochemical oxygen demand (BOD), chemical oxygen demand (COD), and total suspended solids (TSS) analyses were performed to evaluate the efficiency of POME fermentation in the presence of *Lysinibacillus* sp. LC 556247 and *Aspergillus flavus*. Total organic carbon (TOC) analysis was conducted to analyze the organic content in samples.

In detail, the objectives of this study were to: (1) utilize the wastes from agro-industrial sector especially palm oil wastes and bio-valorize into value-added product of biomass fuel with high CEV, and simultaneously (2) reduce the waste accumulated in the palm oil factory.

## 2. Materials and methods

### 2.1. Microorganisms and inoculum preparation

Two types of isolated bacteria and fungus strains *viz*., *Lysinibacillus* sp. LC 556247 and *Aspergillus flavus* were used in this study isolated from POME as reported in our previous study [17]. The genomic DNA (gDNA) of an isolated strain was extracted using Nucleospin® Tissue (Takara, Otsu, Japan). Upon completion of the gDNA extraction, the region containing the 16S rRNA gene was amplified using the universal bacterial primers, 27F (50-AGAGTTTGATCCTGGCTCAG-30) and 1492R (50-GGCTACCTTGTTACGACTT-30), and gDNA using Q5 high-fidelity DNA polymerase (New England BioLabs, MA, USA) by polymerase chain reaction (PCR). The nucleotide sequence of the 1.5-kb amplified fragment, separated on 0.8% agarose gel electrophoresis, was determined by Eurofins Genomics (Tokyo, Japan). The nucleotide sequences of the 16S rRNA gene of the *Lysinibacillus* sp. LC 556247 strain of POME was deposited in the DDBJ/EMBL/GenBank database, under the accession number, LC556247. The 16S rRNA gene sequence similarities were searched using the BLAST programs in the National Centre for Biotechnology Information (NCBI) database. The nucleotide sequences of the 16S rRNA gene of the Bacillaceae were obtained from NCBI. The multiple alignments with the Clustal W program, and the construction of a phylogenetic tree with a maximum likelihood algorithm (bootstrap repeat, 1000), were performed using the MEGAX program version 10.1.8 [17]. Whereas the fungus, *Aspergillus flavus*, was identified by its morphological characteristics at the School of Biological Sciences, Universiti Sains Malaysia (USM) [21]–[24].

*Lysinibacillus* sp. LC 556247 were grown in sterile Nutrient broth (NB) (Brand: HiMedia) overnight in an incubator shaker with 180 rpm agitation speed at 35 ± 2 °C. Upon reaching optical density (OD) 0.6–0.8 at 620 nm wavelength, the bacteria were inoculated (5% w/v) into fermentation medium.

*Aspergillus flavus* was cultured on potato dextrose agar (PDA) (Brand: HiMedia) for 5–7 consecutive days at 35 ± 2 °C incubation temperature prior to spore harvesting using the standard spore suspension method. The spores were counted by direct microscopic counting using a haemocytometer. Serial dilution method was then employed to fix the concentration of the fungus to 1 x 10^6^. The fungus was consequently inoculated (5% w/v) into the fermentation medium.

### 2.2. Feedstocks

POME samples from de-oiling tank and EFB were both collected from United Oil Palm (UOP), Nibong Tebal, Pulau Pinang, Malaysia (geographical coordinates at 5°09′22.3″ N and 100°30′32.3″ E) was used as the feedstock in this study. POME sample from de-oiling tank were selected due to its considerably high oil content as compared to other stages during pond treatment. Both samples were placed at room temperature and the EFB long fibres were shredded (Seng Hup Engineering, Malaysia) and grinded (Daya Korban, Malaysia) to produce short single fibre within 5–10 cm. Preceding experiment, POME was diluted as per one-to-one dilution using distilled water to reduce its high viscosity. Moisture content (MC) of both diluted POME and EFB were evaluated to calculate the ratio of fermentation medium of 7:3, consisting 300 mL of POME and 18 g of EFB and transferred into one litre shake flask.

### 2.3. Shake flask batch fermentation

The fermentation was conducted under three different conditions: (1) Group 1: autoclaved POME and EFB without addition of any microorganisms, (2) Group 2: autoclaved POME and EFB with the addition of *Lysinibacillus* sp. LC 556247 (5% w/v) and *Aspergillus flavus* (5% w/v), and (3) Group 3: non-sterile POME and EFB as it is. These fermentation treatments differ from each other due to the following reasons: (1) Group 1 serves as the control of the study, (2) Group 2 is conducted to elucidate the fermentation effects and the organic compound degradation mechanisms in the presence of *Lysinibacillus* sp. LC 556247 and *Aspergillus flavus*, and (3) Group 3 is to characterize the fermentation effects by the existing consortium of microorganisms and the organic compound degradation mechanisms, as well as enhancement of CEV in its natural condition.

**Figure 1.**
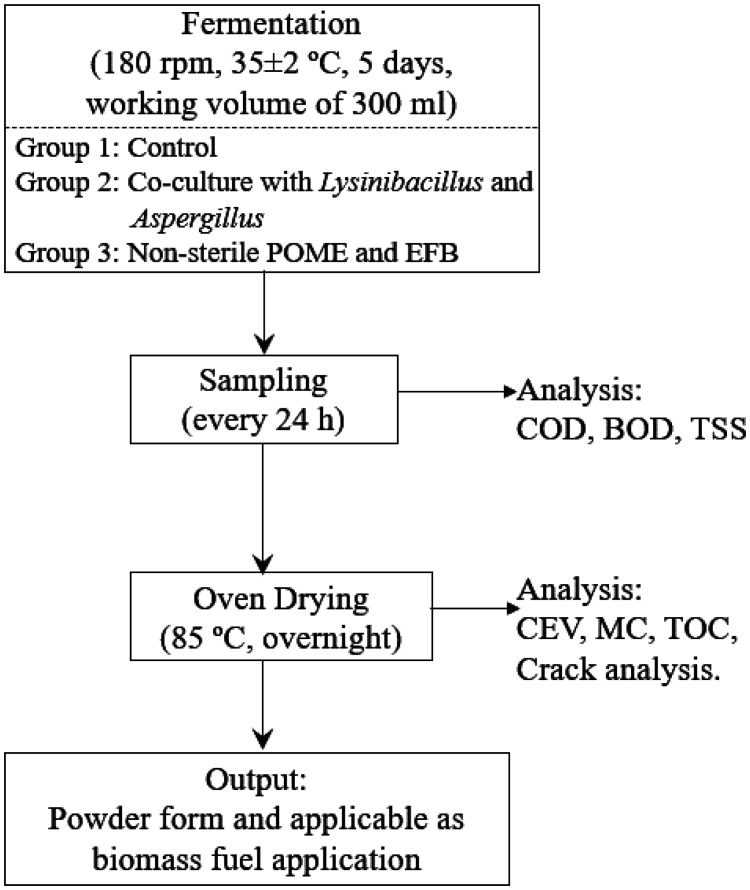
Flow-chart of the overall methodology in this study.

Figure 1 delineates the flow-chart of the overall methodology conducted in this study. The fermentation was carried out with an agitation speed of 180 rpm at 35 ± 2 °C for 5 consecutive days. A total volume of 50 mL sample was collected every 24 hours interval (0, 24, 48, 72, 96, 120 hours) whereby 40 mL of the taken samples were oven dried overnight at 85 °C using an industrial size oven (Binder World FD056UL, Binder GmbH, Tuttlingen, Germany) until constant weight was observed and then subjected for further analyses. The remaining 10 mL were subjected to COD, BOD, and TSS analyses.

### 2.4. Analytical methods and characterization of POME

The fermented and dried samples were homogenized using mortar and pastel prior determination of the CEV and moisture content (MC) analyses. The remaining fermented liquid samples were used for the following analyses of COD, BOD, and TSS. The MC of dried samples was analysed by a moisture analyzer (Sartorius,Germany) in which the setting pre-set for the temperature was 105 °C. The CEV was determined by using an oxygen bomb calorimeter (Parr™ 6200, Fisher Scientific International Inc., Pittsburg, USA), using approximately 0.5–0.6 g of homogenized dried samples similar to that of the method employed by Mohammad et al. [17]. The organic contents present in the fermentation feedstocks were quantify by measuring the COD, BOD and TSS using APHA methods [25]. For COD analysis, 5220 D procedure of closed reflux with a colorimetric method was adopted to evaluate the organic contents in the fermentation feedstocks and was then tabulated as COD removal efficiency, by using Eq 1 [26]. Whilst, the method adopted for BOD analysis was BOD_5_, 5210 B procedure.

$$COD\;Removal\;Efficiency\left(\%\right)=\frac{COD_{i}-COD_{f}}{COD_{i}}x\;100.$$ (Eq 1)

Whereby, COD_i_ is initial COD value and COD_f_ is final COD value. All tests were performed in triplicate to confirm the reproducibility.

One g of homogenized dried sample from each group was weighed using an electronic weighing scale (Mettler Toledo AL 204) and recorded as initial weight. The weighed sample was then transferred into pellet press (Model: Parr, USA). The sample was pressed and compacted into a pellet with the dimension of 12 mm diameter and 8 mm height. The pellet was subjected for crack analysis whereby each pellet was dropped from 1 m high onto the floor. The cracks and final weight of the sample was observed and recorded.

Total Organic Carbon (TOC) analysis has become an important parameter used to monitor overall levels of organic compounds. In this study, 1 g powdered samples with the highest CEV from Group 2 was subjected for analysis to measure the level of organic compounds using TOC analyzer. This analysis was performed by using a Shimadzu TC 5050 Carbon Analyzer (Shimadzu Corporation, Japan). Carbon analyzer are mainly comprised of two different components system; Solid Sample Module-5000 A (SSM) and Total Organic Carbon, (TOC-L) analyzer. Initially, 1 g of homogenous solid samples was weighed and deposited into the SSM with following parameters; furnace temperature of 900 °C and 200 °C for TC and IC content, respectively. The SSM was equipped with purified oxygen at 200 kPa and the carrier gas flow was set at 20 mL/min. Then, the samples were further analyzed using TOC-L analyzer, with furnace temperature set at 680 °C and supplied with 200 kPa purified air. The carrier gas flow was set at 20 mL/min. The TOC was measured in terms of percentage by using the following Eq 2:

Total Carbon(%)=Total Organic Carbon(%)+Inorganic Carbon(%) (Eq 2)

The data were analysed by SPSS Version 26. All results were expressed based on triplicate determinations. A one-way ANOVA (α = 0.05) was used to analyze the data, and a value of less than 0.05 was considered significant.

## 3. Results and discussion

### 3.1. Valorization of POME and EFB as a potential biomass fuel

In this research, diluted POME and EFB were utilized with the ratio of 7:3, which serves as the co-culture fermentation medium or feedstocks for *Lysinibacillus* sp. LC 556247 and *Aspergillus flavus*. Our previous study evinced low CEV for the samples with high ratio of EFB. Thus, in this study, the ratio of POME is increased and the EFB are reduced in order to produce high quality of biomass fuel with enhance CEV [17]. The fermentation process was carried out under aerobic conditions. The utilised POME was obtained from the deoiling tank from palm oil production factory.

**Figure 2.**
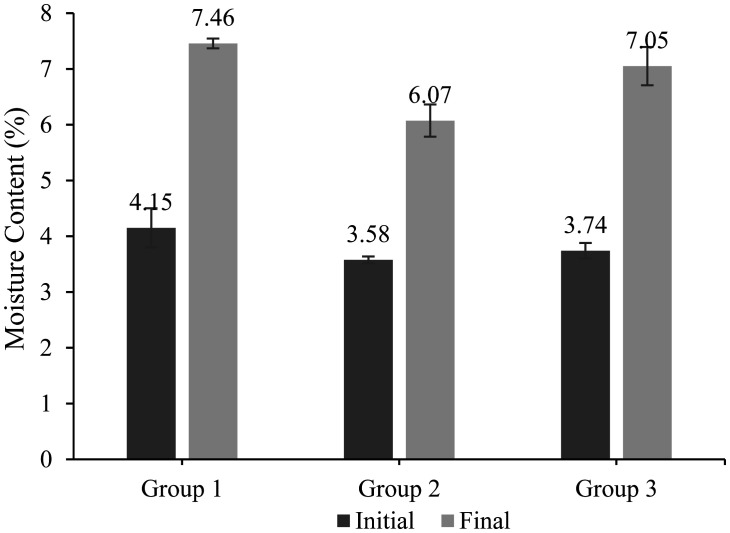
Moisture content (MC) for initial and final samples after 5 days of fermentation and drying process. *Note: Group 1: autoclaved POME without any addition of microbial strain population, Group 2: autoclaved POME with the addition of *Lysinibacillus* sp. LC 556247 (5% w/v) and *Aspergillus flavus* (5% w/v), and Group 3: non-sterile POME (as it is). Means ± standard deviations, *n* = 3.

Figure 2 shows the moisture content (MC) for initial and final samples after 5 days of fermentation and drying process. One of the most essential characteristics of a biomass pellet is the MC. This is due to the fact that MC has a significant effect on the CEV of the biomassic materials [27],[28]. Referring to Figure 2, initially the MC of all fermentation conditions was below 5% in which Group 2 portrayed the lowest moisture (3.58%) compared to Group 1 and Group 3 which are 4.15% and 3.74%, respectively. The moisture of these dried samples was increasing to 7.46% (Group 1), 6.07% (Group 2) and 7.05% (Group 3). Overall, MC for all fermentation conditions were below 10%, upon 5 days of fermentation and drying process, which is applicable for the biomass fuel production [17]. A one-way ANOVA was performed to compare the effect of various sampling time on MC in different groups, which indicate a statistically significant difference.

**Figure 3.**
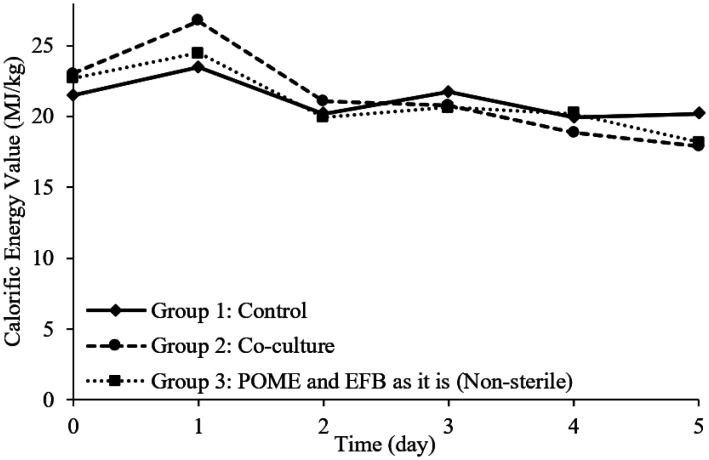
CEV throughout 5 days of fermentation in various fermentation medium condition.

Figure 3 deliniates the CEV changes throughout 5 days in various fermentation condition. The CEV of a biomass fuel is define as the amount of energy generated upon burning the pellet. High quality of biomass fuel indicated by high CEV [17]. The highest CEV obtained in this study was from Group 2 after 1 day of fermentation with the value of 26.71 MJ/kg, which is much higher as compared to other reported CEV of POME (16.99 MJ/kg) and EFB (18.88 MJ/kg) [17]. This observation pinpointing that after 24 hours of treatment in the presence of both *Lysinibacillus* sp. LC 556247 and *Aspergillus flavus* in Group 2, the CEV increase significantly as compared to Group 1 and Group 3. All fermentation conditions indicated similar trend whereby CEV increment are observed from initial reading to first day of fermentation followed by decreasing trend from day one onwards until the end of experiment. Withal, comparison among all three groups shows the highest CEV are obtained after 24 hours of fermentation. The highest CEV obtained from Group 1 and Group 3 were 23.45 MJ/kg and 24.49 MJ/kg, respectively. It is interesting to note that the CEV of Group 1 portray almost no changes from the initial until day 5 of fermentation.

High quality of biomass fuel preferably to consists of MC less than 10% and high CEV [17]. In this study, the highest CEV of 26.17 MJ/kg were obtained throughout valorization of POME and EFB with the ratio of 7:3 in the presence of both *Lysinibacillus* sp. LC 556247 and *Aspergillus flavus* with the final MC of 6.07%. High MC will impose undesirable incomplete combustion, which will lead to obtain low CEV [17]. In addition, it is reported in few studies that individual POME and EFB expressed lower CEV of 16.99 MJ/kg and 18.88 MJ/kg, respectively [16]–[18]. It is also worth noting that the high combustible component such as volatile matter and fixed carbon in EFB may be significant in resulting the high amount of CEV. In another findings, the volatile matter of EFB can reached up to 83.86% and fixed carbon at 18.3% while the incombustible components were relatively low [20].

### 3.2. Characterization and biodegradation of organic compound by robust microorganisms

In addition to evaluating the potential utilization of POME and EFB as biofuel, the effects of robust microorganisms were also performed to study their effects on POME treatment. The biodegradation of organic compounds in wastewater was measured by employing wastewater quality analyses which are chemical oxygen demand (COD), biochemical oxygen demand (BOD), and total suspended solids (TSS). Table 1 delineates the current standards pre-requisites by Department of Environment (DOE), Malaysia and the obtained data in this study.

**Table 1.** Current standard discharge limit set by DOE [29] and data obtained in this study.

Parameters	Standard discharge limit [29]	This study		
		Group 1	Group 2	Group 3
BOD (mg/L)	100	NA	NA	NA
BOD removal efficiency (%)	NA	NA	61.11	48.60
COD removal efficiency (%)	NA	0-5	48.78	36.00
Oil and grease (mg/L)	5	NA	NA	NA
pH	5–9	NA	NA	NA
Temperature (ºC)	45	35 ± 2 °C	35 ± 2 °C	35 ± 2 °C
Initial TSS (mg/L)	NA	13900	13200	13500
Final TSS (mg/L)	400	13800	8300	5400
TSS removal efficiency (%)	NA	0.72	37.12	60.00

*Note: NA: Not Available

**Figure 4.**
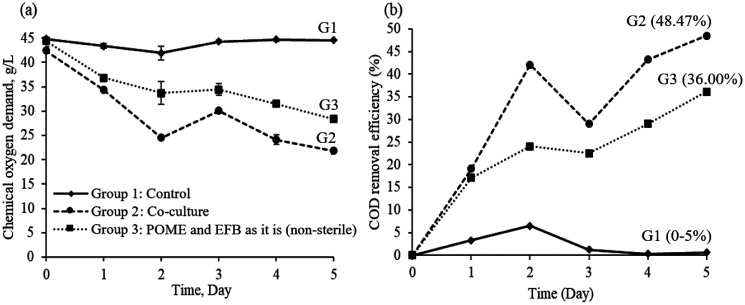
Batch fermentation performance: (a) Chemical oxygen demand (COD) concentration profile, and (b) COD removal efficiency profile in various fermentation condition.

In this study, the COD analysis was adopted to measure the effectiveness of utilising both *Lysinibacillus* sp. LC 556247 and *Aspergillus flavus* co-culture in biodegradation of organic compounds present in POME simultaneously utilising it as their carbon sources. The COD concentration level from initial fermentation until the end experiment was represented in the Figure 4(a) in which COD levels were measured at every 24-hour interval. Referring to Figure 4(a), both fermentation group which has microorganisms shared similar decreasing trend compared to the sterile POME and EFB fermentation medium in Group 1. Howbeit, comparing Group 2 and Group 3, it is noticeable that both *Lysinibacillus* sp. LC 556247 and *Aspergillus flavus* are remarkable in degrading organic components present in the fermentation medium as compared to non-sterile medium. The role of microorganisms, especially robust microorganisms are needed to facilitate degradation of organic matters in a medium [30].

The obtained COD concentration profile were then replotted to determine its removal efficiency as depicted in Figure 4(b). The highest COD removal was recorded with the value of 48.47% in Group 2 at the fifth day of fermentation in the presence of *Lysinibacillus* sp. LC 556247 and *Aspergillus flavus*. Whilst Group 2 fermentation conditions showed the highest efficiency of COD removal, howbeit, Group 3 with non-sterile fermentation medium condition only able to contribute *circa* 36% COD removal at the fifth day of fermentation. This may be due to the nature of non-sterile medium condition whereby a consortium of microorganisms exists in the medium, thus, some inhibitor microbes may deter the degradation of organic compounds in fermentation medium [31]. COD removal efficiency for Group 3 indicate almost no changes throughout five days of experiment.

Biochemical oxygen demand (BOD) is a measure of the dissolved oxygen required by biological organisms to break down the organic material in a particular water sample [32]. The method employed for this analysis was 5210 B BOD for 5 days BOD test, which is approved by the EPA. BOD of the sample was measured by using HANNA Instruments HI98193 Dissolved Oxygen and BOD meter. The obtained BOD removal efficiency was delineated in Table 1. Referring to Table 1, the highest BOD removal efficiency are 61.11% (Group 2) and followed by 48.60% (Group 3). It is worth noting that the BOD level are directly affected by the population of microorganisms that are actively consumed oxygen in a water sample [33]. Furthermore, the EFB were shredded and grinded into short and single fibers will contribute to the increase in the surface are for the decomposition and other physical fluid properties. Therefore, the fermentation efficiency may be affected by the method of cutting fibers.

The TSS was also determined to observe the degrading ability of robust *Lysinibacillus* sp. LC 556247 and *Aspergillus flavus* strains. TSS, is a wastewater analysis primarily to determine the concentration of insoluble organic matter present in the sample interest [34]. In this experiment, TSS was conducted for each fermentation group, prior fermentation and final day of fermentation, as delineated in Table 1. Referring to Table 1, TSS value for all initial sample prior fermentation ranging from 13200–13900 mg/L. Throughout the fermentation, it is noticeable that Group 3, non-sterile fermentation condition exhibited the most degradation of suspended matter followed by Group 2, with the presence of *Lysinibacillus* sp. LC 556247 as well as *Aspergillus flavus* populations and lastly Group 1. This observation indicates that after five consecutive days of aerobic fermentation condition, Group 3 has the highest degradability of insoluble organic constituents in the mixture of POME and EFB medium compared to Group 2 with *Lysinibacillus* sp. LC 556247 as well as *Aspergillus flavus* populations. In conventional wastewater treatment method, a second stage treatment is required to further reduce the TSS. Howbeit, in this study, through drying process, all moisture will be removed and suspended solid will remain. The output of powder samples was pelletized and, in this way, an zero wastewater discharge treatment are achievable.

It is worth noting the degradation mechanism of both bacteria as well as fungus are comparably different. The degradation of cellulose as well as hemicellulose into carbohydrates metabolites are easily facilitated by aerobic bacteria, thereby carbohydrate metabolites such as nucleotide sugar, fructose and mannose, starch, sucrose as well as butanoate were produced [29]. *Lysinibacillus* sp. LC 556247 is identified as facultative anaerobic bacteria, hence, it can divert to anaerobic digestion mode under oxygen-deficient ambient in the fermentation medium in which the fermentation products with more than two carbon atoms, for example, alcohols and aromatic fatty acids are converted into acetate as well as hydrogen [30]. It is also further clarified in another research that products from hydrolysis stage was converted into butyric acid, propionic acid, ethanol, acetic acid, carbon dioxide and hydrogen by the bacteria [28],[29]. Whilst, *Aspergillus* sp. is able to accumulate lipids in large amount by using POME as its substrates [33]. Apart from accumulating lipids, *Aspergillus* sp. is also known as lignin degrader whereby lignin as well as hemicellulose were degraded into cellulose, hence resulted in cellulose accumulation in which much more simpler to degrade by microorganism [34].

### 3.3. Biomass fuel pellet compactness analysis

Crack analysis was conducted by dropping pellets from 1 m high to characterize the compactness of the pellets. Table 2 delineates the weight differences of the pellets before and after dropped from 1 m high. This analysis was tested only for the dried sample with the highest CEV from each fermentation group.

**Figure 5.**
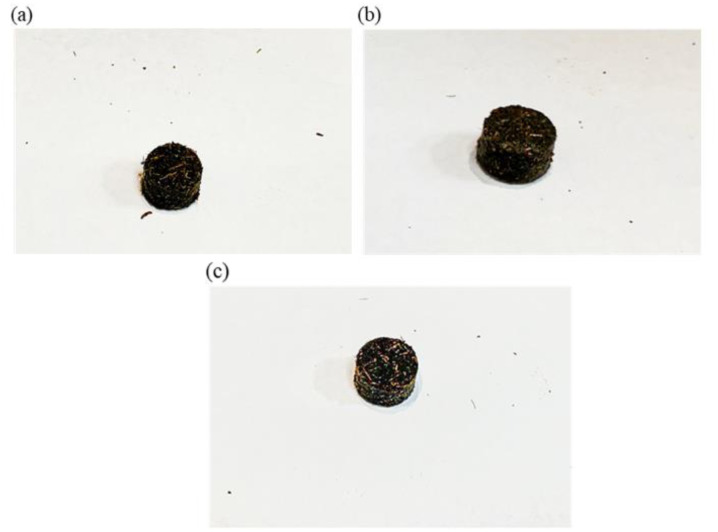
Photographs of pellets after dropped: (a) Group 1, (b) Group 2, and (c) Group 3.

Referring to Table 2, the largest weight difference exhibited from Group 1 followed by Group 2 and Group 3. Apart from the weight difference, the pellet specks were also noticeable after conducting the experiment where Group 1 has the most specks surrounding the pellet. The following pictures in Figure 5 shows the photographs of pellet condition after dropped.

**Table 2.** Weight differences of the pellets before and after dropped from 1 m high in crack analysis.

Sample name	Initial weight (g)	Weight after dropped (g)	Weight difference (g)
Group 1 Day 1	1.0885	1.0267	0.0618
Group 2 Day 1	1.0853	1.0422	0.0431
Group 3 Day 1	1.0843	1.0477	0.0366

It is worth noting that the pellet stability and compactness may be affected by the presence of oil as mentioned by Hassan et al. in their study, whereby high POME ratio indicated the most stable as well as expanded the least due to its oily material [15]. From another perspective, the presence of binders also gave significant affects in terms of the durability of the fuel pellet in which the higher the amount of binder, the stronger the fuel pellet [35],[36]. Correlating to this study, EFB was mixed with POME as part of fermentation medium, hence, become the natural binders of the fuel pellet. As a result, all fermentation conditions portray less cracks and weight difference below 0.07 g. This may be because EFB are fibres rich in cellulose (makes up to 65%), lignin (up to 29.2%), hemicellulose (up to 28.8%), and extractive (3.7%). The hydrogen bonding between different layers of cellulose chains, and along the cross-linking of lignin with both cellulose and hemicellulose, resulted in a complex web of links that contributes to the structural strength of EFB fibres [11]. Hence, the stability and durability of the pellet were improved with the presence of both lignin as the natural binder [27],[28] and POME oily materials.

### 3.4. Total Organic Carbon (TOC), Total Inorganic Carbon (TIC), and Total Carbon (TC)

The sample with the highest CEV of 26.71 MJ/kg from Group 2 are subjected for analysis of organic compounds. The percentage values obtained for TOC is 64.67%, TC is 64.68%, and TIC is 0.00869%.

## 4. Conclusion

The reduction of COD values was reported as the results of the microorganism reactions in biodegradation process. *Aspergillus flavus* and *Lysinibacillus* sp. are among the identified microorganisms that were proliferate and at the same time causes the reduction in COD concentration of POME. The highest CEV of 26.71 MJ/kg were obtained by mixing POME and EFB with the ratio of 7:3 and sufficiently co-cultured for only one day. This study successfully achieved zero waste water discharge. Valorization of palm oil wastes into value added product is one of the sustainable and environmental-friendly approaches to produce useful products and reduce the waste. Thus, it is crucial that this research work can be further explored to determine the economic viability and practicality to implement at industrial scale as a stand alone or require second treatment stage in palm oil waste treatment. This strategy is aligned with the aim of UNESCO Sustainable Development Goal Number 12 to ensures sustainable consumption and production patterns.
